# Cognitive Development of Infants Exposed to the Zika Virus in Puerto Rico

**DOI:** 10.1001/jamanetworkopen.2019.14061

**Published:** 2019-10-25

**Authors:** Viviane Valdes, Carmen D. Zorrilla, Laurel Gabard-Durnam, Natalia Muler-Mendez, Zarin Ibnat Rahman, Diego Rivera, Charles A. Nelson

**Affiliations:** 1 Laboratories of Cognitive Neuroscience, Boston Children’s Hospital, Harvard Medical School, Boston, Massachusetts; 2 Obstetrics and Gynecology, Maternal-Infant Studies Center, University of Puerto Rico School of Medicine, Medical Sciences Campus, San Juan, Puerto Rico; 3 Harvard Graduate School of Education, Cambridge, Massachusetts

## Abstract

**Importance:**

Existing research has established a causal link between Zika virus (ZIKV) infection and severe birth defects or consequent health impairments; however, more subtle cognitive impairments have not been explored.

**Objective:**

To determine whether infants of mothers with at least 1 positive ZIKV test show differences in cognitive scores at ages 3 to 6 months and ages 9 to 12 months.

**Design, Setting, and Participants:**

This cross-sectional study recruited infants enrolled in existing ZIKV study cohorts associated with the Maternal-Infant Studies Center and the Puerto Rico Clinical and Translational Research Consortium at the University of Puerto Rico and from the broader San Juan metropolitan area. The study took place at the Puerto Rico Clinical and Translational Research Consortium at the University of Puerto Rico. Participants were recruited through convenience sampling if their mothers underwent ZIKV testing prenatally and were at the target ages during the study period. Infants who were born preterm (<36 weeks’ gestational age), with low birth weight (<2500 g), or with a known genetic disorder were excluded. Infants were tested from ages 3 to 6 months or ages 9 to 12 months from May 2018 to April 2019. Data analysis was performed from March to April 2019.

**Exposures:**

Zika virus status was measured prenatally and in the early postnatal period using real-time polymerase chain reaction or a ZIKV IgM antibody capture enzyme-linked immunosorbent assay.

**Main Outcomes and Measures:**

The infants’ development was assessed using the Mullen Scales of Early Learning (translated to Spanish and adapted for Puerto Rico), and assessors were blinded to each infant’s ZIKV status.

**Results:**

A total of 65 study participants were included. The mean (SD) age of the infants at the time of cognitive testing was 8.98 (3.19) months. Most of the infants were white (55 [84.6%]) and Puerto Rican (64 [98.5%]); 38 of the infants were male (58.5%). General cognitive and domain-specific scores did not differ significantly between prenatally ZIKV-positive and ZIKV-negative infants except for receptive language score (mean difference = 5.52; *t* = 2.10; *P* = .04). Exposure to ZIKV (*B* = −5.69; β = −0.26 [95% CI −11.01 to −0.36]; *P* = .04) and a measure of Hurricane Maria exposure (time without water, *B* = −0.05; β = −0.27 [95% CI, −0.10 to −0.01]; *P* = .03) were both independently and significantly associated with receptive language scores after adjusting for key confounders.

**Conclusions and Relevance:**

Although infants exposed to ZIKV prenatally showed unaffected motor and visually mediated cognitive development, they did show deficits in receptive language scores. Receptive language skills were also associated with the degree of exposure to Hurricane Maria, with those who spent more time without water after the hurricane having lower receptive language scores.

## Key Points

**Question:**

Is prenatal maternal exposure to Zika virus (ZIKV) associated with cognitive scores among infants after adjusting for confounders, including demographic characteristics, socioeconomic status, maternal mental health, and exposure to Hurricane Maria?

**Findings:**

In this cross-sectional study of 65 Puerto Rican infants aged 3 to 12 months, those with prenatal ZIKV exposure had lower receptive language scores. Exposure to ZIKV and Hurricane Maria both were significantly and inversely associated with receptive language scores.

**Meaning:**

Even among infants without microcephaly or congenital Zika syndrome, prenatal maternal ZIKV infection is associated with lower receptive language scores during the first year of life; however, exposure to ZIKV does not appear to be associated with other domains of cognitive development.

## Teaser

This cross-sectional study of 65 infants in Puerto Rico examines the associations of exposure to maternal Zika virus and Hurricane Maria with infant cognitive scores.

## Introduction

After the Zika virus (ZIKV) was first identified in 1947 in rhesus macaques in the Zika forest near Kampala, Uganda, there were a few rare and isolated cases in humans.^1^ However, in 2007, an outbreak was reported in the Federated States of Micronesia during which 73% of the population was infected over a 2-year period, leading the World Health Organization and Centers for Disease Control and Prevention (CDC) to recognize the outbreak as a pandemic.^1,2^ By 2013, there were 294 confirmed cases in French Polynesia over a 3-month period, and the outbreak only worsened over the next few years.^3^

The CDC^4^ defines ZIKV as a flavivirus that is transmitted by *Aedes* species mosquitoes and can cause fever, rash, and joint pain. In addition to these milder symptoms, ZIKV infection early in pregnancy has been causally linked to abnormalities in brain structure, including microcephaly.^5,6,7,8^ Beyond the higher incidence of microcephaly observed in newborns exposed to ZIKV during fetal development, ZIKV has also been associated with ophthalmologic complications and neural abnormalities, such as thin or atrophied cerebral mantle (gray matter), absence of the cavum septum pellucidum, shortened corpus callosum, dilated ventricles, and prominent choroid plexus (which forms cerebral spinal fluid).^9,10,11,12^ The latest reports^13^ (from 2018) suggest that, in US territories, among 1450 children of mothers with laboratory evidence of confirmed or possible ZIKV infection, 6% of children had at least 1 identified ZIKV-associated birth defect, 9% had 1 identified ZIKV-associated neurodevelopmental abnormality, and 1% had both.

Infection with ZIKV in utero is also associated with a number of health impairments after birth. A study^14^ in Brazil of 19 children aged 19 to 24 months with confirmed congenital ZIKV infection found that 11 experienced seizures, 4 experienced retinal abnormalities, 10 experienced sleeping difficulties, 9 experienced feeding difficulties, 13 had impaired responses to auditory stimulus, 11 had impaired responses to visual stimuli, 15 had severe motor impairment, and 14 had cerebral palsy. Given the emerging evidence of an association between ZIKV and atypical brain development in utero, severe birth defects, and consequent health impairments,^8^ research is needed to examine how ZIKV exposure is associated with subsequent physical and cognitive development over the first year of life.^15,16^

In light of the findings of an association between ZIKV exposure and alterations in brain development, in the current study, we sought to examine the possible functional consequences of such alterations—specifically, whether there is an association between ZIKV status and cognitive, language, and motor development. We did so by recruiting a sample of ZIKV-exposed infants in San Juan, Puerto Rico. The CDC^4^ reported that, as of July 7, 2016, there were 5582 confirmed cases in Puerto Rico, with potentially many more unconfirmed cases in patients without symptoms. By May 20, 2017, more than 40 000 confirmed cases of ZIKV infection had been diagnosed in Puerto Rico.^17,18^ Of these, 3703 were women infected during pregnancy, and there were at least 38 cases of ZIKV-associated birth defects in Puerto Rico alone.^17,18^ However, concerns have been raised about the reliability of these data, with public health experts asserting that ZIKV-affected births and ZIKV cases were underreported, especially after Hurricane Maria affected much of the country’s infrastructure in September 2017 and ZIKV testing was halted.^19,20^ Within this Puerto Rican population, this study aimed to address the following questions. First, do infants of mothers who have at least 1 positive ZIKV test prenatally or postnatally show differences in developmental assessment scores at ages 3 to 6 months and ages 9 to 12 months? Second, are measurements of demographic characteristics, socioeconomic status, maternal mental health, and Hurricane Maria exposure (ie, time without resources after Hurricane Maria) associated with ZIKV status or cognitive scores during the first postnatal year?

## Methods

### Study Design

Infants and mothers for the current study were recruited through a convenience sampling method from the Zika in Infants and Pregnancy study (ClinicalTrials.gov identifier: NCT02856984),^21^ other ZIKV studies at the Puerto Rico Clinical and Translational Research Consortium, and from the broader San Juan, Puerto Rico, community. The Zika in Infants and Pregnancy study is a National Institutes of Health–sponsored international observational prospective cohort study assessing the strength of the association between ZIKV infection during pregnancy and adverse maternal and/or fetal outcomes. The study prospectively enrolled a cohort of pregnant women during their first and early second trimester of pregnancy and followed them through their pregnancies to identify ZIKV through blood testing.^21,22^

 Ethical approval for the study was obtained from the institutional review boards at Boston Children’s Hospital and the University of Puerto Rico, Medical Sciences Campus. Written informed consent was obtained for all infant-mother dyads who took part in the study. This study follows the Strengthening the Reporting of Observational Studies in Epidemiology (STROBE) reporting guideline.

We had planned to implement the current study during 2017, but before implementation, Hurricane Maria made landfall in Puerto Rico on September 20, 2017. The study was halted until the infrastructure was adequate and participants were able to be enrolled and travel to the study site. Hurricane Maria, a category 5 storm, had a direct path over the island on September 20, 2017. Despite all the preparations and assumptions that the infrastructure would stand winds of 90 to 150 mph and gusts of 175 mph, the island’s power, water, and communications infrastructure, which were weakened by years of poor maintenance and previous damage from Hurricane Irma, completely collapsed. In this context, pregnant women experienced disruption of prenatal care and monitoring, regardless of trimester. This disruption could have been critical to those at risk because of medical and social complications. It is known that stress related to natural disasters has a long-term impact on both mothers and infants. Maternal prenatal stress related to natural disasters affects fetal growth and is associated with younger gestational age, lower birth weights, and children’s cognitive and language development.^22,23^

In the current study, we recruited mothers who underwent confirmed ZIKV testing during pregnancy, who had a viable pregnancy, and who had an infant who was aged 3 to 6 months or aged 9 to 12 months (±2 weeks) during our study period from May 2018 to April 2019. We chose to focus on these age groups because we were interested in development during the first year of life and wanted to maximize our sample size. To be included, infants’ mothers also had to have ZIKV testing available from at least 1 time point during their pregnancy (either positive or negative ZIKV IgM polymerase chain reaction [PCR] results). Infants who were born preterm (<36 weeks of gestation), with low birth weight (<2500 g), or with a known genetic disorder (including microcephaly and congenital Zika syndrome) were excluded from the sample to avoid confounding. Exceptions to the birth weight allowance were made in 1 case for a set of twins (birth weight >2300 g) who did not have a neonatal intensive care unit stay after their delivery and who met all other eligibility criteria. A total of 65 participants of either ages 3 to 6 or 9 to 12 months were included in the current study.

Zika virus infection status (positive or negative) was determined through prenatal and, in some cases, additional postnatal maternal blood testing. The 2 diagnostic tools used were the Trioplex real-time (RT) PCR assay (CDC) or the ZIKV IgM antibody capture enzyme-linked immunosorbent assay (MAC-ELISA) (CDC).^24,25^ Trioplex RT-PCR assay results are able to identify ZIKV, dengue, or chikungunya viral RNA. The test is able to detect infection in blood samples from the acute phase of infection up to 14 days after the onset of symptoms. Consequently, positive results suggest a current infection. The ZIKV MAC-ELISA is able to presumptively identify IgM antibodies to ZIKV. Levels of IgM can vary but generally are positive starting near day 4 after the onset of symptoms and continuing for 12 or more weeks after initial infection. Some research^10^ has also noted prolonged viremia in pregnancy, potentially due to viral replication in the fetus or placenta, which may harbor the virus. One concern with IgM testing for diagnosis is related to cross-reactivity with prior flavivirus infections, such as dengue and chikungunya viruses.

For a participant to be considered ZIKV positive, the mother had to have had at least 1 prenatal or postnatal Trioplex RT-PCR result that was positive or a ZIKV-specific MAC-ELISA with a result of positive or presumptive positive. The MAC-ELISA testing for dengue and chikungunya viruses was also available to rule out whether the participants were actually positive for one of those viruses and not ZIKV specifically. Mothers were tested with a serological assay with emergency use authorization approval from the US Food and Drug Administration. Mothers who had all negative test results at all time points were considered negative, and those with multiple negative results and 1 equivocal result on the ZIKV MAC-ELISA were considered negative. This approach has been taken in past research on prenatal ZIKV.^26^ The number of time points for ZIKV testing ranged from 1 to 14 (mean = 6.23 time points with multiple types of tests conducted at each time point) from as early as 4 weeks’ gestational age to 40 days post partum.

### Measures of Cognitive and Motor Development

The Mullen Scales of Early Learning (MSEL) tool was used to measure cognitive (including fine motor, receptive language, expressive language, and visual reception) and gross motor development. This laboratory assessment tool has been validated for use with children ranging in age from a few months to 6 years, which facilitates longitudinal research in developmental populations.^27^ In our sample, the MSEL was administered for infants aged 3 to 6 and 9 to 12 months (±2 weeks). The MSEL is a standardized assessment of child cognitive functioning that uses 5 subscales to capture developmental trajectories: gross motor, visual reception, fine motor, receptive language, and expressive language. The cognitive composite score can be reflected as a cognitive T score sum, which is the sum of each scale’s T scores or as a standard score (reference population mean [SD], 100 [15]). Each of the 5 scales also has a T score with a reference population mean (SD) of 50 (10).

The test, which was administered by a trained research assistant in the hospital, uses different standardized tasks to engage the child using props (eg, rubber balls, triangles, whistle, doll, or mirror). These tasks increase incrementally in difficulty and required developmental level. The administrator records scores on various scales (eg, 0-1 or 0-5) on the basis of the child’s response to stimuli and computes raw scores by adding up points within each category. A standardized chart is used to determine age equivalencies, T scores, confidence intervals, percentile ranks, developmental stages, and descriptive categories and to compute an early learning composite score for each child.

The MSEL has been shown to have good construct validity, feasibility, and sensitivity in low-income settings, and translated versions of the assessment are consistent with those used in English-speaking areas.^27^ For the current study, the MSEL protocol was followed by bilingual administrators in English, and the verbal prompts were translated to Spanish. These translations were completed by a bilingual research assistant and revised by 4 additional research assistants and 1 of the principal investigators to ensure that they were accurate and culturally appropriate. The administrators were blinded to the infant’s ZIKV status. For the current study, early learning composite scores (also referred to as cognitive standard scores) that measure overall cognitive development, as well as domain-specific T scores, were used in the analyses. Both the overall cognitive standard score and the domain-specific scores were age normalized to allow for comparisons across infants with different ages.

Demographic characteristics were also included in the current study to better characterize our sample and determine whether they were mediating any observed associations between ZIKV status and cognitive scores at ages 3 to 6 months and 9 to 12 months. These included key demographic characteristics, such as infant birth weight (continuous, pounds), infant sex (binary, male or female), race (binary, white or black), ethnicity (binary, Puerto Rican or Dominican), infant’s age at the time of testing (continuous, months), and primary language spoken at home (binary, Spanish or bilingual).

Socioeconomic status was also measured through maternal and paternal education levels (categorical, less than high school, high school or general equivalency diploma, associate’s degree, or bachelor’s degree or higher), maternal and paternal employment status (categorical, full-time employment, part-time employment, or unemployed or works at home), household size (continuous, number of people living in household), home status (categorical, owned, rented, living without paying rent, or public housing), annual income (categorical, <$5000, $5000-$11 999, $12 000-$15 999, $16 000-$24 999, and $25 000-$34 999), and nonparental care (continuous, number of hours that the child was cared for by a nonparent weekly).

Maternal mental health was assessed using the Generalized Anxiety Disorder–7 Questionnaire,^28^ the Patient Health Questionnaire–9,^29^ the Edinburgh Postnatal Depression Scale,^30^ and the Perceived Stress Scale.^31^ Versions of these tools that were translated to Spanish and culturally validated in other Latin American countries were used in the current study.^32,33,34,35,36,37,38^ All of the maternal mental health composite scores were treated continuously in bivariate analyses.

Exposure to Hurricane Maria was measured using 3 variables: number of days spent without electricity, water, or cell phone service after Hurricane Maria made landfall in Puerto Rico in September 2017. All of the covariates listed were assessed during the infant study visit starting in May 2018 (8 months after Hurricane Maria made landfall in Puerto Rico) and were collected through maternal self-report. Hurricane exposure (time without resources after Hurricane Maria) was assessed given the existing body of literature linking natural disaster exposure to maternal mental health and child cognitive development. The infants in the current study may have been particularly affected by maternal prenatal stress, because Hurricane Maria landed in Puerto Rico while 55 of 65 of them were in utero. Of the 29 ZIKV-exposed infants, 25 were in utero during Hurricane Maria.

### Statistical Analysis

Statistical analysis was performed using SPSS statistical software version 24 (IBM). Initial exploratory analysis included generation of descriptive statistics to assess for nonnormality in the variables of interest. Independent samples *t* tests were conducted to determine whether there were differences in MSEL T scores by domain (gross motor, fine motor, visual reception, receptive language, and expressive language) and cognitive standard score when compared between ZIKV-positive and ZIKV-negative participants. Next, Pearson χ^2^ (including Cramér V), analysis of variance, and bivariate correlations were calculated to determine whether any of the covariates of interest was associated with ZIKV status or cognitive scores. Finally, an ordinary least squares linear regression model (including standardized coefficients) was used to determine whether ZIKV status was significantly associated with cognitive scores after adjusting for key demographic characteristics (infant birth weight, infant sex, infant race, infant ethnicity, infant age at the time of testing, and primary language spoken at home) and any other covariates of interest that were significantly associated with ZIKV status or cognitive scores at the bivariate level. A final multivariate regression model was used to determine whether there was an interaction effect between ZIKV status and time without resources following Hurricane Maria on cognitive scores. Time without resources was mean centered before creating the interaction term. All variables included in the regression model had complete data. For all analyses, a priori levels of significance were set at *P* < .05 and were 2-tailed. Data analysis was performed from March to April 2019.

## Results

### Participant Characteristics

In total, 65 participants were included in our analyses, 36 of whom (55.4%) were categorized as having negative ZIKV status and 29 of whom (44.6%) were categorized as having positive ZIKV status. Of 29 infants, 28 were identified through serological testing and 1 was identified through molecular testing. Three of 29 mothers had symptoms of ZIKV.

The mean (SD) infant age at the time of cognitive testing was 8.98 (3.19) months. Most of the infants were white (55 [84.6%]) and Puerto Rican (64 [98.5%]). Thirty-eight of the infants were male (58.5%) and 27 were female (41.5%). Most infants (60 [92.3%]) lived in a household where Spanish was the primary language, and the remainder lived in a bilingual household. Twenty-two mothers (33.8%) had a bachelor’s degree or higher, followed by an associate’s degree (19 [29.2%]), high school or general equivalency diploma (20 [30.8%]), and less than high school (4 [6.2%]). Most fathers (25 [38.5%]) had an associate’s degree, followed by high school or general equivalency diploma (21 [32.3%]), less than high school (10 [15.4%]), and bachelor’s degree or higher (6 [9.2%]). Most mothers were unemployed or worked at home (34 [52.3%]), and most fathers were employed full time (42 [64.6%]). Table 1 provides full descriptive statistics for all covariates.

**Table 1.** Descriptive Statistics for Sample Demographic Characteristics and Covariates, by ZIKV Status

Variable	ZIKV Negative (n = 36)	ZIKV Positive (n = 29)
Infant birth weight, mean (SD), kg	15.51 (2.44)	15.97 (2.55)
Infant sex, No. (%)		
Male	22 (61.1)	16 (55.2)
Female	14 (38.9)	13 (44.8)
Infant race, No. (%)		
White	32 (88.9)	23 (79.3)
Black	4 (11.1)	6 (20.7)
Infant ethnicity, No. (%)		
Puerto Rican	36 (100)	28 (96.6)
Dominican	0	1 (3.4)
Infant age at testing, mean (SD), mo	9.29 (2.95)	8.61 (3.48)
Primary language, No. (%)		
Spanish	32 (88.9)	28 (96.6)
Bilingual (English and Spanish)	4 (11.1)	1 (3.4)
Maternal education, No. (%)		
Less than high school	2 (5.6)	2 (6.9)
High school or general equivalency diploma	11 (30.6)	9 (31)
Associate’s degree	13 (36.1)	6 (20.7)
Bachelor’s degree or higher	10 (27.8)	12 (41.4)
Paternal education, No. (%)		
Less than high school	7 (19.4)	3 (10.3)
High school or general equivalency diploma	9 (25)	12 (41.4)
Associate’s degree	14 (38.9)	11 (37.9)
Bachelor’s degree or higher	4 (11.1)	2 (6.9)
Missing	2 (5.6)	1 (3.4)
Maternal employment, No. (%)		
Full time	11 (30.6)	5 (17.2)
Part time	4 (11.1)	11 (37.9)
Unemployed or works at home	21 (58.3)	13 (44.8)
Paternal employment, No. (%)		
Full time	23 (63.9)	19 (65.5)
Part time	5 (13.9)	3 (10.3)
Unemployed or works at home	5 (13.9)	5 (17.2)
Missing	3 (8.3)	2 (6.9)
Household size, mean (SD), No. of members	3.92 (1.25)	3.90 (1.42)
Home status, No. (%)		
Owned	16 (44.4)	5 (17.2)
Rented	8 (22.2)	11 (37.9)
Occupied for free	4 (11.1)	6 (20.7)
Public housing	8 (22.2)	7 (24.1)
Nonparental care, mean (SD), h/wk	18.22 (20)	25.88 (23.52)
Maternal anxiety score on Generalized Anxiety Disorder–7 Questionnaire, mean (SD)	6.4 (5.23)	5.31 (5.16)
Maternal depression score on Patient Health Questionnaire–9, mean (SD)	7.46 (5.71)	5.72 (4.68)
Maternal depression score on Edinburgh Postnatal Depression Scale, mean (SD)	5.97 (5.87)	5.21 (3.77)
Maternal Perceived Stress Scale score, mean (SD)	16.89 (6.02)	14.41 (5.51)
Time without electricity after Hurricane Maria, mean (SD), d	138.17 (71.82)	146.34 (73.73)
Time without water after Hurricane Maria, mean (SD), d	22.96 (46.52)	25.31 (68.87)
Time without cell phone service after Hurricane Maria, mean (SD), d	60.10 (64.11)	72.59 (88.21)

Abbreviation: ZIKV, Zika virus.

### Cognitive Scores and ZIKV Status

The mean (SD) cognitive standard score for the full sample was 105.01 (13.13), which is 0.35 SD above the population mean of 100. For the full sample, mean (SD) T scores were 46.82 (11.14) for gross motor score (*z* score = −0.32), 56.66 (12.57) for fine motor score (*z* score = 0.67), 57.78 (10.02) for visual reception score (*z* score = 0.78), 46.95 (10.83) for receptive language score (*z* score = −0.31), and 48.35 (9.41) for expressive language score (*z* score = −0.17). For the ZIKV-negative infants, the mean (SD) cognitive T score was 214.14 (28.58) and the mean (SD) cognitive standard score was 107.22 (14.14). For the ZIKV-positive infants, the mean (SD) cognitive T score was 204.28 (23.01) and the mean (SD) standard score was 102.28 (11.42) (Table 2).

**Table 2.** Descriptive Statistics for Participant Cognitive Scores, by ZIKV Status

Variable	ZIKV Negative (n = 36)	ZIKV Positive (n = 29)
Cognitive T score, mean (SD)	214.14 (28.58)	204.28 (23.01)
Cognitive standard score, mean (SD)	107.22 (14.14)	102.28 (11.42)
Cognitive descriptive categories of infants, No. (%)		
Very low	0	0
Below average	2 (5.6)	1 (3.4)
Average	21 (58.3)	23 (79.3)
Above average	13 (36.1)	5 (17.2)
Very high	0	0
Gross motor T score, mean (SD)	47.19 (10.59)	46.34 (11.96)
Gross motor descriptive categories of infants, No. (%)		
Very low	2 (5.6)	1 (3)
Below average	5 (13.9)	7 (24.1)
Average	25 (69.4)	16 (55.2)
Above average	4 (11.1)	3 (10.3)
Very high	0	0
Fine motor T score, mean (SD)	57.61 (13.53)	55.48 (11.38)
Fine motor descriptive categories of infants, No. (%)		
Very low	1 (2.8)	0
Below average	3 (8.3)	2 (6.9)
Average	12 (33.3)	19 (65.5)
Above average	15 (41.7)	7 (24.1)
Very high	5 (13.9)	1 (3.4)
Visual reception T score, mean (SD)	57.36 (11.08)	58.28 (8.69)
Visual reception descriptive categories of infants, No. (%)		
Very low	0	0
Below average	2 (5.6)	1 (3.4)
Average	21 (58.3)	17 (58.6)
Above average	8 (22.2)	9 (31)
Very high	5 (13.9)	2 (6.9)
Receptive language T score	49.42 (10.98)	43.90 (9.98)
Receptive language descriptive categories of infants, No. (%)		
Very low	2 (5.6)	3 (10.3)
Below average	5 (13.9)	6 (20.7)
Average	23 (63.9)	20 (69)
Above average	6 (16.7)	0
Very high	0	0
Expressive language T score, mean (SD)	49.75 (8.02)	46.62 (10.79)
Expressive language descriptive categories of infants, No. (%)		
Very low	0	3 (10.3)
Below average	3 (8.3)	3 (10.3)
Average	30 (83.3)	23 (79.3)
Above average	3 (8.3)	0
Very high	0	0

Abbreviation: ZIKV, Zika virus.

**Figure 1.**
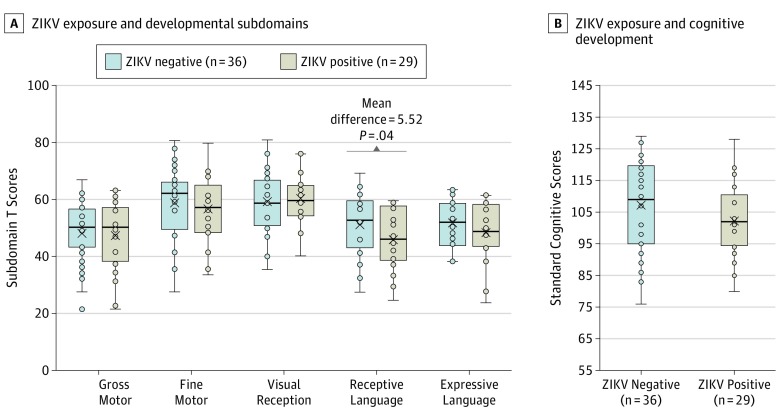
Zika Virus (ZIKV) Exposure and Cognitive Scores A, Associations between ZIKV exposure and developmental subdomains are shown. B, Associations between ZIKV exposure and cognitive development are shown. Center horizontal lines indicate medians; X, means; top and bottom borders of boxes, first and third quartiles; and whiskers, minimum and maximum values, not including outliers.

Domain-specific and composite cognitive scores did not differ significantly across ZIKV-positive and ZIKV-negative groups in independent samples *t* tests except in the receptive language domain (Figure 1). Infants who tested negative for ZIKV prenatally had a mean (SD) score of 49.42 (10.98), and those who tested positive for ZIKV prenatally had a mean (SD) score of 43.90 (9.98) (mean difference = 5.52; *t* = 2.10; *P* = .04).

### Associations Between ZIKV Status, Receptive Language, and Selected Covariates

**Figure 2.**
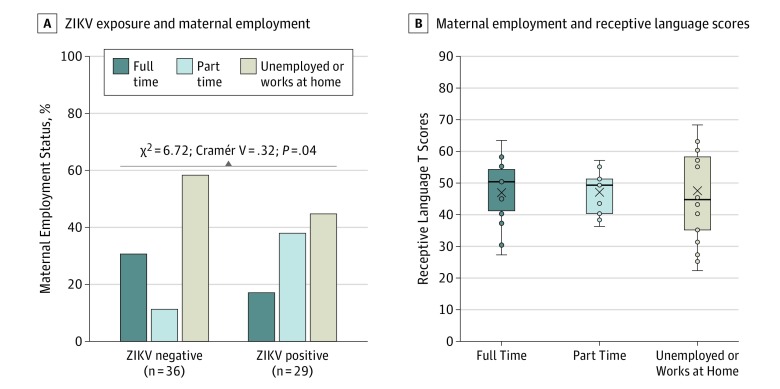
Associations Between Zika Virus (ZIKV) Exposure, Maternal Employment, and Receptive Language Scores A, Associations between ZIKV exposure and maternal employment status are shown. B, Associations between maternal employment and receptive language scores are shown. Center horizontal lines indicate medians; X, means; top and bottom borders of boxes, first and third quartiles; and whiskers, minimum and maximum values, not including outliers.

Pearson χ^2^ was used to determine whether there was an association between maternal ZIKV status and education (maternal and paternal), maternal employment, paternal employment, and home status. None of the associations was significantly associated except for ZIKV status and maternal employment (χ^2^ = 6.72; Cramér V = 0.32; *P* = .04) (Figure 2). One-way analysis of variance was conducted to determine whether group means for household size, annual income, hours in nonparental care, maternal anxiety, maternal depression (Edinburgh Postnatal Depression Scale and Patient Health Questionnaire–9), maternal perceived stress, and time without resources after Hurricane Maria (electricity, water, and cell phone service, independently) differed by ZIKV status. None of the associations was statistically significant (eTable 1 in the Supplement).

The same process was undertaken to determine whether any covariates (aside from ZIKV status) had significant associations with receptive language scores. Spearman correlations were calculated to determine whether receptive language scores were significantly associated with maternal education, paternal education, maternal employment, paternal employment, annual income, and home status. None of the associations was statistically significant. Pearson correlations were also calculated to determine whether receptive language scores were significantly associated with household size, hours in nonparental care, maternal anxiety, maternal depression (Edinburgh Postnatal Depression Scale and Patient Health Questionnaire–9), maternal perceived stress, and time without resources after Hurricane Maria (electricity, water, and cell phone service, independently). None of the associations was significantly associated except for number of days without access to water and receptive language scores (*r =* −0.275; *P* = .03) (eTable 1 and eFigure in the Supplement).

### Multivariate Regression Analysis: ZIKV Status and Cognitive Scores Adjusting for Covariates

A linear regression model was used to determine whether ZIKV status was significantly associated with receptive language T scores after adjusting for infant birth weight, infant sex, infant race, infant ethnicity, infant age at the time of testing, primary language spoken at home, maternal employment, and days without water after Hurricane Maria. In this model, days without water (*B* = −0.05; β = −0.27 [95% CI, −0.10 to −0.01]; *P* = .03) and ZIKV status (*B* = −5.69; β = −0.26 [95% CI, −11.01 to −0.36]; *P* = .04) were significantly associated with receptive language scores (Table 3). That is, prenatal exposure to ZIKV and more days without water after Hurricane Maria were significantly and independently associated with lower receptive language scores. No interaction effects were found between ZIKV and number of days without access to water (eTable 2 in the Supplement).

**Table 3.** Multivariate Regression Model: ZIKV Status and Receptive Language Scores, Adjusted for Covariates

Variable	Unstandardized Coefficient (SE)^a^	Standardized Coefficients (95% CI)	*P* Value
Infant birth weight, lb	0.86 (1.18)	0.09 (−1.51 to 3.23)	.47
Infant sex, male or female	−1.11 (2.76)	−0.05 (−6.65 to 4.42)	.69
Infant race, white or black	−3.54 (3.90)	−0.12 (−11.36 to 4.28)	.37
Infant ethnicity, Puerto Rican or Dominican	4.83 (11.18)	0.06 (−17.58 to 27.24)	.67
Infant age, mo	−0.82 (0.42)	−0.24 (−1.67 to 0.03)	.06
Primary language, Spanish or bilingual	2.75 (4.90)	0.07 (−7.07 to 12.58)	.58
Mother’s employment, full time, part time, or unemployed or worked at home	0.49 (1.63)	0.04 (−2.77 to 3.75)	.76
Hurricane exposure, d without water	−0.05 (0.02)	−0.27 (−0.10 to −0.01)	.03^b^
ZIKV status, negative or positive	−5.69 (2.66)	−0.26 (−11.01 to −0.36)	.04^b^

Abbreviation: ZIKV, Zika virus.

^a^ Constant (SD) = 45.86 (19.52); *R*^2^ = 0.22; adjusted *R*^2^ = 0.09; *F* *=* 1.71; *df* = 9.

^b^ *P* < .05, 2-tailed.

## Discussion

The objectives of the current study were to determine whether infants of mothers who had at least 1 positive prenatal ZIKV test result show differences in cognitive scores from ages 3 to 6 months and 9 to 12 months and whether ZIKV status was associated with scores on cognitive domains after adjusting for confounders. We found that, although ZIKV-exposed infants had unaffected motor and visually mediated cognitive development, they did show modest deficits in language in the first year of life. Receptive language skills were also associated with the degree of exposure to Hurricane Maria (indexed by time without water after the hurricane), but these factors affected language scores independently. These findings suggest that exposure to ZIKV prenatally, even in the absence of microcephaly, can have significant associations with language development within the first year of life and may lay the foundations for future effects later in development, because language skills tend to build on each other.

The current sample of 29 infants prenatally exposed to ZIKV and 36 unexposed controls had mean (SD) cognitive standard scores of 105.01 (13.13), which is about 0.35 SD above the population mean of 100 on the MSEL. This slightly higher score may be reflective of our study’s small sample size or high levels of maternal education in our sample.^39^ Although the cognitive standard score was higher than expected, this was not reflected in domain-specific scores, where scores for some categories of cognition (ie, gross motor, receptive language, and expressive language) were lower than those for the reference population. When examining whether scores in each of these domains differed by ZIKV exposure groups, only receptive language scores differed significantly between ZIKV-positive and ZIKV-negative groups. Given that all other domains on the MSEL were not associated with ZIKV exposure, it is difficult to interpret the association with receptive language. For example, without magnetic resonance imaging data on these infants (none of whom had microcephaly), it is difficult to know whether language-related areas of the brain were selectively affected by viral exposure.

Furthermore, although past research^9,12,40,41,42,43,44,45^ has extensively reported ophthalmologic manifestations associated with congenital Zika syndrome, the current study did not identify visual reception differences in the first year of life at the behavioral level. This could be because infants with ZIKV exposure but without congenital Zika syndrome or microcephaly are at less risk for ophthalmologic complications or because the behavioral consequences of these complications may emerge later in development. Future research later in development and using nonbehavioral methods such as electroencephalography would be useful to make this distinction. It is also possible that other areas of cognition less tied to early visual-motor coordination may show impairments later as infants develop, particularly because the MSEL tests a limited form of early cognition, and early language problems indicate the potential for other cognitive skills to be affected. Conversely, it is also possible that ZIKV exposure has little association with cognitive development unless such exposure is associated with microcephaly or other structural brain abnormalities.^46,47,48^

Although, to our knowledge, there is no research on the link between ZIKV exposure and cognitive outcomes, there is some evidence of an association between the experience of natural disasters and cognitive outcomes. This research^23,49,50,51^ suggests that exposure to natural disasters is linked to lower cognitive and, specifically, language functioning in toddlers. These studies^23,49,50^ found that both productive (expressive) and receptive language scores are significantly different between toddlers (aged 2 years) of mothers exposed and not exposed to natural disasters prenatally. Some authors^52,53,54^ also propose that elevated maternal glucocorticoids levels after exposure to stressful events may account for differences observed in language development, as well as internalizing problems from ages 2 to 3 years.

### Limitations

The present analysis has a number of limitations that should be considered. One is that the use of cross-sectional, observational data to characterize potentially predictive factors does not allow us to support claims about causality between ZIKV exposure and cognitive outcomes in infancy. Another is that, given the challenge of recruiting mothers who had ZIKV testing throughout their pregnancy and after the experience of a major traumatic natural disaster (Hurricane Maria) where infrastructure was significantly affected, our sample size is small, so we may have missed associations with smaller effect sizes linking ZIKV exposure and early physical and cognitive development during the first year. Notably, the maternal education levels in this study were not reflective of the general population, because many of the mothers in our study had higher levels of education (93.8% with high school level education or higher) compared with census findings in San Juan (79.6% with high school level education or higher).^55^ In addition, our study used IgM testing in some mothers to identify ZIKV infection, which, according to the CDC, cannot be used alone to confirm ZIKV infection and requires further testing.^56^ However, all participants were also tested for dengue virus and chikungunya virus antibodies and, in the presence of a positive result for these tests, they were not considered ZIKV positive.

## Conclusions

The current study may provide early evidence that exposure to ZIKV prenatally, in the absence of severe outcomes such as microcephaly, is not associated with observed impairments in motor skills, expressive language, and visual reception. Infants with prenatal ZIKV exposure only displayed early behavioral associations with receptive language in the first year of life, although larger sample sizes are needed to determine whether this association is clinically significant. This study also provides additional evidence to an existing body of research on exposure to natural disasters and cognitive development. Our findings suggest that, as early as age 3 months and within the first year of life, hurricane exposure (specifically, time without access to water after Hurricane Maria) is associated with lower receptive language scores. Future research should aim to replicate these findings in larger samples and new contexts. Additional studies should also examine these associations beyond the first year of life to determine whether the association with receptive language is observed and whether there are associations with other domains later in development. In addition, clinicians should assess cognitive development in ZIKV-exposed infants and children and provide interventions that can target deficits early.

## Notes

Supplement. **eTable 1.** Associations Between ZIKV, Receptive Language, and Selected Covariates **eFigure.** Association Between Hurricane Maria Impact and Receptive Language Scores **eTable 2.** Multivariate Regression Model: Interaction Between Hurricane Impact and ZIKV on Receptive Language Scores, Adjusting for Covariates
