# Supplementary material for: Cognitive Development of Infants Exposed to the Zika Virus in Puerto Rico
Source: JAMA Netw Open. 2019 Oct 25;2(10):e1914061. doi: 10.1001/jamanetworkopen.2019.14061 (PMC6822087; doi:10.1001/jamanetworkopen.2019.14061)
Supplement: Supplement. — eTable 1. Associations Between ZIKV, Receptive Language, and Selected Covariates eFigure. Association Between Hurricane Maria Impact and Receptive Language Scores eTable 2. Multivariate Regression Model: Interaction Between Hurricane Impact and ZIKV on Receptive Language Scores, Adjusting for Covariates [file jamanetwopen-2-e1914061-s001.pdf]

Supplementary Online Content

Valdes V, Zorrilla CD, Gabard-Durnam, L, et al. Cognitive development of infants exposed to the Zika virus in Puerto Rico. *JAMA Netw Open*. 2019;2(10):e1914061. doi:10.1001/jamanetworkopen.2019.14061

eTable 1. Associations Between ZIKV, Receptive Language, and Selected Covariates
eFigure. Association Between Hurricane Maria Impact and Receptive Language Scores
eTable 2. Multivariate Regression Model: Interaction Between Hurricane Impact and ZIKV on Receptive Language Scores, Adjusting for Covariates

This supplementary material has been provided by the authors to give readers additional information about their work.

© 2019 Valdes V et al. *JAMA Network Open.*

eTable 1. Associations Between ZIKV, Receptive Language, and Selected Covariates

	ZIKV status (negative/positive)	Receptive language t-scores
ZIKV status (negative/positive)	--	--
Receptive language t-scores	F = 4.40*	--
Maternal education (less than high school, high school/GED, associate's, bachelor or higher)	$\chi2$ = 2.23<br>Cramer's V = 0.19	Rho = 0.09
Paternal education (less than high school, high school/GED, associate's, bachelor or higher)	$\chi2$ = 2.50<br>Cramer's V = 0.20	Rho = 0.02
Maternal employment (fulltime, part-time, unemployed/housework)	$\chi2$ = 6.72<br>Cramer's V = 0.32*	Rho = 0.05
Paternal employment (fulltime, part-time, unemployed/housework)	$\chi2$ = 0.28<br>Cramer's V = 0.07	Rho = -0.24
Home status (owned, rented, occupied for free, public housing)	$\chi2$ = 6.02<br>Cramer's V = 0.30	Rho = -0.06
Household size (number of people living in household)	F = 0.004	r = 0.14
Annual income (ordinal from low to high)	F = 0.17	Rho = 0.07
Hours in non-parental care	F = 2.01	r = -0.23
Maternal anxiety	F = 0.70	r = 0.15
Maternal depression (EPDS)	F = 0.37	r = -0.05
Maternal depression (PHQ)	F = 1.71	r = 0.13
Maternal perceived stress	F = 2.89	r = 0.17
Days without electricity	F = 0.20	r = -0.02
Days without water	F = 0.03	r = -0.28*
Days without cell service	F = 0.44	r = -0.15

*p < 0.05, two-tailed

© 2019 Valdes V et al. *JAMA Network Open.*

eFigure. Association Between Hurricane Maria Impact and Receptive Language Scores

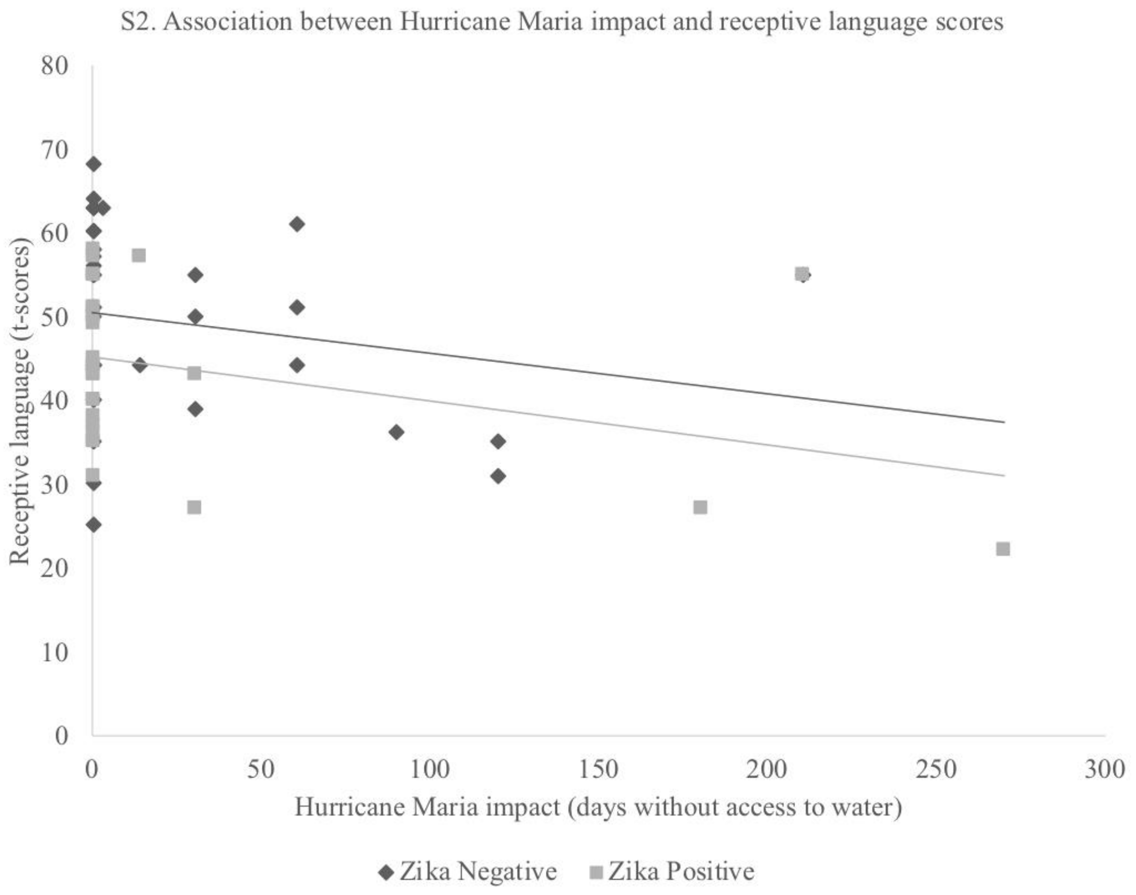

© 2019 Valdes V et al. *JAMA Network Open.*

eTable 2. Multivariate Regression Model: Interaction Between Hurricane Impact and ZIKV on Receptive Language Scores, Adjusting For Covariates (n = 65)

	Unstandardized coefficients (standard error)	Standardized coefficients	95% Confidence interval	P-value
Infant birthweight (in lbs)	0.91 (1.22)	0.10	-1.54, 3.36	0.46
Infant sex (male/female)	-1.13 (2.79)	-0.05	-6.72, 4.46	0.69
Infant race (white/black)	-3.46 (3.96)	-0.12	-11.39, 4.48	0.39
Infant ethnicity (Puerto Rican/Dominican)	4.65 (11.32)	0.05	-18.04, 27.33	0.68
Infant age (in months)	-0.82 (0.43)	-0.24	-1.68, 0.04	0.06
Primary language (Spanish, bilingual)	2.81 (4.95)	0.07	-7.12, 12.74	0.57
Mother’s employment (fulltime, part-time, unemployed/housework)	0.48 (1.64)	0.04	-2.81, 3.77	0.77
Hurricane impact (days without water)	-0.05 (0.04)	-0.24	-0.12, 0.03	0.25
Zika status (negative/positive)	-5.70 (2.68)*	-0.26	-11.07, -0.32	0.04
Hurricane impact x zika status	-0.01 (0.05)	-0.05	-0.11, 0.09	0.83
Constant	45.36 (19.83)			
R2	0.22			
Adjusted R2	0.08			
df for model F	9			
F	1.52			

*p < 0.05, two-tailed

© 2019 Valdes V et al. *JAMA Network Open.*
